# Isavuconazole Pharmacokinetics in Critically Ill Patients: Relationship with Clinical Effectiveness and Patient Safety

**DOI:** 10.3390/antibiotics13080706

**Published:** 2024-07-29

**Authors:** María Martín-Cerezuela, Cristina Maya Gallegos, María Remedios Marqués-Miñana, María Jesús Broch Porcar, Andrés Cruz-Sánchez, Juan Carlos Mateo-Pardo, José Esteban Peris Ribera, Ricardo Gimeno, Álvaro Castellanos-Ortega, José Luis Poveda Andrés, Paula Ramírez Galleymore

**Affiliations:** 1 Pharmacy Unit, Hospital Universitario y Politécnico La Fe, 46026 Valencia, Spain; martin_marcer@gva.es (M.M.-C.); marques_mre@gva.es (M.R.M.-M.); cruz_and@gva.es (A.C.-S.); poveda_josand@gva.es (J.L.P.A.); 2 Intensive Care Unit, Hospital Universitario y Politécnico La Fe, 46026 Valencia, Spain; mcmayagallegos@gmail.com (C.M.G.); ixuscorreu@gmail.com (M.J.B.P.); juancarlosmateopardo@gmail.com (J.C.M.-P.); ricardogimeno55@gmail.com (R.G.); castellanos_alv@gva.es (Á.C.-O.); 3 Department of Pharmaceutics, Faculty of Pharmacy, University of Valencia, 46100 Valencia, Spain; jose.e.peris@uv.es

**Keywords:** isavuconazole, pharmacokinetics, critical care, extracorporeal membrane oxygenation, antifungal

## Abstract

Isavuconazole is used to treat fungal infections. This study aims to describe isavuconazole pharmacokinetics in critically ill patients and evaluate their relationship with clinical efficacy and patient safety. We conducted a prospective, observational study in patients treated with intravenous isavuconazole. Samples were collected at predose (Cmin), 1 h (Cmax) and 12 h (C50) after the last dose. The plasma concentration was determined by high-performance liquid chromatography. The relationship between plasma concentration and clinical and microbiological outcomes and safety was evaluated. The influence of covariates (age, sex, weight, SAPS3, creatinine, liver enzymes and extracorporeal devices: continuous renal replacement therapy (CRRT) and extracorporeal membrane oxygenation (ECMO)) was analysed. Population pharmacokinetic modelling was performed using NONMEN^®^. A total of 71 isavuconazole samples from 24 patients were analysed. The mean Cmin was 1.76 (1.02) mg/L; 87.5% reached the optimal therapeutic target and 12.5% were below 1 mg/L. Population pharmacokinetics were best described by a one-compartment model with first-order elimination. No factor had a significant impact on the plasma concentration or pharmacokinetic parameters. Thus, isavuconazole could be safely used in a critically ill population, even in those treated with CRRT and ECMO, from a pharmacokinetic standpoint. Therefore, routine therapeutic drug monitoring may not be strictly necessary in daily clinical practice.

## 1. Introduction

The impact of the appropriateness rate and the degree of delay in treatment initiation on patient prognosis has been extensively studied in bacterial and fungal infections [1]. More recently, the pharmacokinetic (Pk) profile has been included as a factor to be considered when prescribing antimicrobials. In critically ill patients, the presence of Pk derangement is a constant finding in all clinical studies [2]. In light of the results, amendments to dosages, interval posology and infusion time, or the need for therapeutic drug monitoring (TDM) have been recommended for many antibiotics when treating severe infections [3]. Moreover, some studies have demonstrated clinical improvement related to Pk optimisation [4,5].

Pk derangement also affects antifungal drugs [6]. Fluconazole has been replaced by echinocandins in the treatment of invasive candidiasis (IC) due to their broader spectrum and more stable Pk profiles [7,8]. Voriconazole was considered the first-line therapy for aspergillosis. However, TDM of voriconazole in critically ill patients has been strongly recommended by international guidelines due to its narrow therapeutic window, non-linear pharmacokinetics and the high inter- and intra-individual variability of blood levels [3]. In fact, low serum levels have been correlated with clinical failure, whereas high serum levels have been associated with hepatic toxicity [4]. Isavuconazole, a broad-spectrum antifungal, has been shown to be non-inferior to voriconazole for the treatment of invasive disease caused by *Aspergillus* spp. and other filamentous fungi [9]. In contrast to voriconazole, isavuconazole has better security and Pk profiles, and a post hoc analysis showed no relationship between drug exposure (specific minimum inhibitory concentration (MIC) values) and outcome parameters [10,11]. Consequently, isavuconazole TDM is not considered necessary [3]. Nevertheless, extrapolating these conclusions to critically ill patients could result in inaccuracies. The limited number of studies on isavuconazole Pk in critical care patients have shown inconsistent results [12,13,14,15]. Impaired liver function, increased volume of distribution (Vd), decreased albumin concentration, continuous renal replacement therapy (CRRT) or extracorporeal membrane oxygenation (ECMO) may alter the pharmacokinetic profile of isavuconazole and make the prediction of drug concentrations difficult, so TDM may be necessary in this population [15,16,17,18,19,20].

This study aims to assess isavuconazole Pk in critically ill patients and its potential association with efficacy and adverse effects. The factors influencing isavunazole Pk in this population will also be evaluated by means of a pharmacokinetic model.

## 2. Methods

### 2.1. Study Population

This prospective observational study included adult patients admitted to a medical Intensive Care Unit (ICU) and treated with intravenous isavuconazole for at least 96 h from January 2023 to December 2023. The study was conducted in a 24-bed ICU of a tertiary university hospital. Our local Ethics Committee, as well as the Spanish Medicines Agency (UMI-CEF-2018-01), approved the study protocol. Informed consent was obtained from all included patients or their legally authorised representatives.

All patients received intravenous isavuconazole at a loading dose of 200 mg q8h for six doses, followed by a maintenance dose of 200 mg q24h; the infusion time was 60 min, in accordance with the established recommendations [9]. Demographic, clinical, microbiological and therapeutic variables were extracted from the electronic medical records systems.

### 2.2. Isavuconazole Pharmacokinetics

Samples were obtained after 48–96 h of isavuconazole treatment. Blood samples were collected in EDTA tubes (7 mL) at predose (Cmin), 1 h (Cmax) and 12 h (C50) after completing the drug infusion. Plasma was obtained after centrifugation (1000 rpm for 10 min) and was stored at −80 °C until analysis. The assay used the reagents and high-performance liquid chromatography (HPLC) column provided by the ChromSystems HPLC Kit for TDM of itraconazole, posaconazole and voriconazole. The isocratic flow rate was set at 1.2 mL/min. Detection was performed using a fluorescence detector with the excitation wavelength set at 261 nm and the emission wavelength set at 366 nm [21].

Although there is no total consensus about the normal range in isavuconazole trough concentrations, most authors accept a range between 1 and 5 mg/L [3,20,22,23]. Population pharmacokinetic parameters were determined based on the obtained plasma concentrations using the computer software NONMEN^®^ (v.5), implementing a first-order conditional estimation method with interaction (ADVAN 1, TRANS 2). Covariate analyses were conducted using a stepwise forward selection procedure. The evaluated variables included age; sex; weight; simplified acute physiologic score (SAPS3); serum creatinine; total bilirubin; the liver enzymes aspartate-aminotransferase, alanine-aminotransferase and gamma-glutamyl transferase (AST/ALT/GGT); albumin; and the use of CRRT and ECMO. The impact of covariates on pharmacokinetic parameters (such as the clearance and the volume of distribution) was initially assessed by visual inspection of the graphical representation of each covariate against the clearance and volume of distribution to evaluate their inclusion in the final model [24]. In each step of forward selection, a univariate analysis of each patient covariate with an observable trend was performed to add the significant covariate to the model. A *p*-value < 0.05 was considered statistically significant. The average area under the concentration–time curve (AUC) for each patient was calculated using the Bayesian posterior parameter estimates from the final model and by using the trapezoidal rule in NONMEN. The AUC was calculated by determining the total dose and dividing it by the clearance for each patient [23,25]. 

### 2.3. Definitions

Invasive candidiasis (IC) was considered to be a result of both bloodstream and deep-seated invasive infections caused by *Candida* species. An IC diagnosis requires a clinical suspicion of infection plus a positive blood culture or other sterile body fluids [7]. Invasive Pulmonary Aspergilosis (IPA) diagnosis required pulmonary infiltrate or nodules, documented by chest computed tomography, or a cavitated infiltrate not attributed to another cause plus mycological evidence (microscopy, culture or galactomannan, alone or in combination) obtained via alveolar lavage [26,27,28]. Mucormycosis diagnosis was based on culturing the organism from normally sterile body locations and/or tissue histology [29].

Treatment was considered empirical in patients with signs and symptoms of infection along with specific risk factors for fungal infection, and without established radiological or microbiological evidence of fungal infection. Treatment was considered targeted in patients with a confirmed fungal infection as a result of culture, histopathology or microscopic examination [7,30]. Clinical response was defined as the resolution of the signs (including radiology) and symptoms present at the time of the infection diagnosis. Microbiological response was defined as the negativisation of the microbiological cultures obtained from the source of the infection at the end of the antimicrobial treatment [7,28,29].

Potential isavuconazole-related adverse effects such as QT abnormalities, neutropenia or hepatotoxicity were observed by daily electrocardiograms (ECGs) and laboratory assessments [9]. Other possible adverse events such as headache, abdominal pain, seizures, nausea or vomiting were evaluated according to medical reports [31]. The appearance of adverse events and their relationship with isavuconazole treatment was classified in accordance with the Common Terminology Criteria for Adverse Events v5.0 (CTCAE, National Cancer Institute, Bethesda, MD, USA) [32].

### 2.4. Statistical Analysis

The exploratory analysis of the Pk parameters and covariates was conducted in R statistical software v3.2.1, and the statistical analysis was performed using Stata^®^ v14.0 software. Categorical variables were summarised as absolute numbers and percentages and analysed with a chi-square test or Fisher’s exact test when indicated. Continuous variables were reported as mean and standard deviation or median and interquartile range (IQR), depending on their homogeneity, and were compared using Student’s *t*-test or the Mann–Whitney U test as appropriate. For all comparisons, a value of *p* ≤ 0.05 was considered statistically significant.

## 3. Results

### 3.1. Patient Characteristics

Over the study period, 24 patients were included in the study. Their demographic and clinical characteristics are depicted in Table 1. Fungal infection was not confirmed in 13 patients (54.2%). Confirmed diagnoses included candidemia (n 7; 6 *Candida auris*, 1 *Candida albicans*), ventriculitis due to *Candida parapsilosis* (n 1), IPA (n 2) and mucormycosis (n 1). Isavuconazole was used as a rescue treatment in three patients, due to previous antifungal therapy failure with voriconazole (n 2) or amphotericin B (n 1). In eight patients (33.3%), isavuconazole was administered in combination with anidulafungin (n 5), amphotericin B (n 2) or caspofungin (n 1). The median duration of isavuconazole treatment was 8 (4–14.5) days.

Clinical response was achieved in 6 of the 11 patients with a confirmed diagnosis (54.5%) and microbiological response was achieved in 8 of the 11 patients with a confirmed diagnosis (72.7%). Global intra-ICU mortality was 58.3% (54.5% and 61.5%, respectively, for patients with and without a confirmed diagnosis).

### 3.2. Isavuconazole Pharmacokinetics

**Figure 1:**
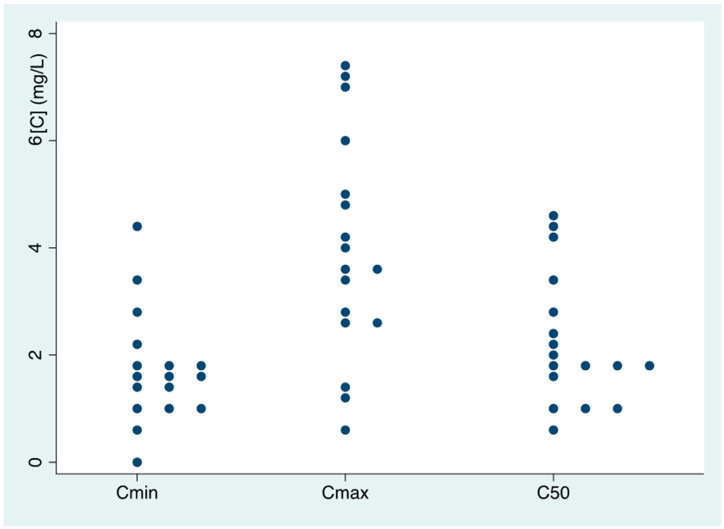
Plasma levels of isavuconazole.

A total of 71 isavuconazole plasma concentrations were available for model development. The mean predose concentration (Cmin) of isavuconazole was 1.76 (1.02) mg/L (median: 1.7 mg/L, range: 0.1–4.5); the mean of Cmax was 3.99 (1.96) mg/L (median: 4 mg/L, range: 0.7–7.4); and the mean C50 was 2.26 (1.20) mg/L (median: 2 mg/L, range: 0.3–4.6). Figure 1 shows the plasma concentrations obtained. Twenty-one patients (87.5%) had Cmin levels within the considered therapeutic interval (1–5 mg/L), three patients (12.5%) were below the interval and no patient was above the interval.

A one-compartment model with first-order elimination was identified as the optimal model to describe the Pk of isavuconazole in this population. None of the studied covariates improved the predictive ability of the model, so no covariates were introduced in the final model. The estimated pharmacokinetic parameters of the isavuconazole model are shown in Table 2.

### 3.3. Relationship between Isavuconazole Pk and Clinical Parameters

No statistically significant Cmin difference was detected between patients who did or did not respond to treatment. Likewise, no relationship was found between trough levels and microbiological response (Table 3).

**Figure 2:**
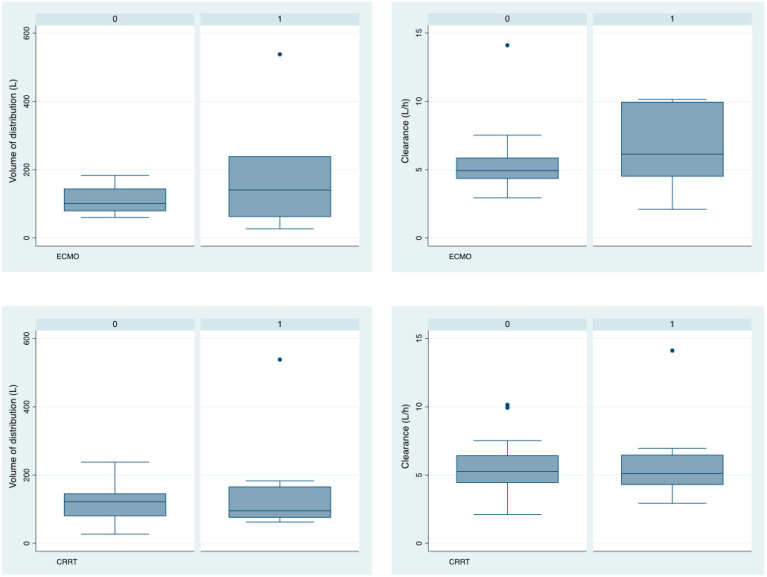
Relationship between ECMO and CRRT and pharmacokinetic parameters (volume of distribution and clearance). CRRT: continuous renal replacement therapy; ECMO: extracorporeal membrane oxygenation; 1: yes; 0: no.

Despite the inter-individual variability, the use of extracorporeal devices did not have a significant impact on isavuconazole population pharmacokinetic parameters (Vd, Cl) (Figure 2). No significant differences were found between the two groups for demographic or clinical variables, except for the SAPS3 score, which showed that patients requiring ECMO support were more severely affected (71.86 (13.83) vs. 56.09 (11.59); *p* = 0.019) and bilirubin (2.06 (1.60) vs. 0.60 (0.61) μmol/L; *p* = 0–016). No significant differences were found between trough plasma levels and CRRT or ECMO support (Table 3). Two patients required support with ECMO and CRRT but were affected at the Cmin, which was 1.1 mg/L in both patients.

Possible adverse events related to isavuconazole administration were only reported in two patients. In one patient, increased bilirubin and grade 1 liver enzymes were observed, and in another patient, a grade 2 liver enzyme increase was observed. In none of the cases with adverse events was there a relationship with isavuconazole plasma concentrations. No relationship was found between higher isavuconazole plasma levels and hepatotoxicity (*p* = 0.779). Six patients (20.8%) had a Cmax > 5 mg/L. However, toxicity did not develop in any of these patients. No patient discontinued treatment due to adverse effects.

## 4. Discussion

Isavuconazole pharmacokinetics were assessed in a well-represented sample of critically ill patients. However, we detected significant variability in trough and peak plasma concentrations; 87.5% of our patients had plasma concentrations within the therapeutic range. Moreover, these values were not associated with any clinical or microbiological outcomes. We did not identify any factor that clearly impacted isavuconazole pharmacokinetics, including the use of CRRT or ECMO. We found that a one-compartment model best predicted isavuconazole concentrations in critically patients, and no covariate was able to improve the model.

While no established pharmacodynamic target has been defined, most authors recommend maintaining a trough isavuconazole concentration > 1 mg/L [33,34]. In randomised clinical trials, 97% of patients achieved trough concentrations > 1 mg/L, and no concentration-dependent relationship was observed for efficacy [23,35]. Trough concentrations were lower in real-world practice patients compared to clinical trials (mean 2.98 ± 1.91 mg/L vs. 3.30 ± 2.18 mg/L; *p* = 0.014); but >90% of real-world samples were above 1 mg/L [34]. Additionally, in the SECURE clinical trial, >97% of patients had a Cmin concentration below 7 mg/L, and no concentration-dependent relationship was observed for toxicity [35]. A threshold of 4.6–5.1 mg/L has been proposed as a safety target to avoid gastrointestinal toxicity [22,31,33]. Therefore, routine isavuconazole therapeutic drug monitoring (TDM) is not considered necessary [3,34,35]. However, TDM has been recommended in specific clinical scenarios, including the treatment of critically ill patients.

Mean isavuconazole trough plasma concentrations in critically ill patients have been shown to be lower than those in the general population [36]. Mikulska et al. found that ICU patients had significantly lower isavuconazole blood levels compared to the non-ICU population. Nevertheless, it should be noted that plasma concentrations in ICU patients were within the therapeutic range [36]. Some factors have been related to lower Cmin levels in critically ill patients: female sex, age ≤ 65 years, body mass index (BMI) > 25, SOFA score > 12 points and use of CRRT or ECMO [13,14,18,36,37]. We evaluated all these variables in our series; however, none of these factors were relevant to our patients’ isavuconazole pharmacokinetic parameters and were excluded as covariates in our compartmental model. In contrast to Höhl et al., even though 58.3% of our patients had a BMI > 25, this variable had no impact on the pharmacokinetics of isavuconazole and was also excluded from the final model [13,14,36].

Although oral isavuconazole administration seems to be better studied by a bi-compartmental model, previous publications agree with our mono-compartmental model in intravenous administration [20]. Moreover, the model by Perez et al. only found CRRT as a significant covariant for isavuconazole Pk in 18 critically ill patients [20].

Hatzl et al. suggest an increase in the loading dose to achieve therapeutic concentrations. Three patients in our series obtained a plasma concentration < 1 mg/L after administration of the standard dose, so this could be considered a future strategy to optimise pharmacokinetics in critically ill patients [38]. Other authors propose an adaptive dosing strategy according to a population model of total and unbound isavuconazole concentrations, but our study design did not account for the assessment of protein binding [15].

The relationship between isavuconazole plasma concentrations and clinical or microbiological endpoints has not been previously assessed specifically in the critically ill population [9,23]. As almost no patients were outside the therapeutic interval, the relationship with the clinical or microbiological response could not have been assessed if lower levels had been present. Our results are concordant with clinical trials, showing no relationship between trough levels and efficacy or toxicity. Probably, in our study, other clinical factors should have been related to the response rate.

The effect of ECMO treatment on drug Pk is a highly relevant topic in the current medical literature. Although some authors have suggested that ECMO could affect isavuconazole Pk [18,19], our study showed no effect on Pk variables or in the compartmental model. This agrees with a study by Mertens et al., in which no direct effect of ECMO on isavuconazole Pk could be corroborated. Likewise, the ECMO treatment was not a covariate in the model of Perez et al., and no significant sequestration of the drug has been demonstrated in the ECMO [12,20,25].

Some limitations should be considered in our study. Firstly, the main limitation is the small number of patients who could be included, as this can limit the generalisability of our findings. Moreover, we did not perform a pharmacokinetic follow-up, which limited our ability to build more complex models or assess additional covariate effects. Additionally, efficacy assessment could only be performed in confirmed fungal infection diagnoses, potentially limiting the statistical power of the sample. We did not evaluate potential drug–drug interactions with concomitant treatments, and we did not differentiate the free and plasma protein-bound fraction for the development of the compartmental model, which may alter the plasma levels of isavuconazole.

In conclusion, our results suggest that isavuconazole could be safely used in the critically ill population, even in those treated with CRRT and ECMO, from a pharmacokinetic standpoint. Moreover, PK parameters were not related to clinical endpoints. Therefore, routine therapeutic drug monitoring may not be strictly necessary in daily clinical practice.

## Figures and Tables

**Table 1:** Clinical and demographic characteristics of patients treated with isavuconazole.

Total (n = 24)	
Cause of ICU Admission	
Septic shock (n, %)	3 (12.5)
Cardiogenic shock (n, %)	4 (16.7)
Acute respiratory failure (n, %)	15 (62.5)
Acute stroke (n, %)	2 (8.3)
Co-morbidities	
Haematological disease	2 (8.3)
Diabetes mellitus, n (%)	5 (20.8)
HIV, n (%)	1 (4.17)
Corticosteroid therapy, n (%)	3 (12.5)
Immunosuppression, n (%) Liver disease, n (%)	4 (16.7) 0 (0.0)
Age (years) (mean, SD)	53.2 (16.4)
Male (n, %)	17 (70.8)
Weight (kg) (mean, SD)	75.1 (12.6)
Height (cm) (mean, SD)	169.7 (0.1)
Body mass index (kg/m^2^) (mean, SD)	26.03 (3.77)
SAPS3 score (median, IQR)	63 (57–71.5)
Mechanical ventilation (n, %)	23 (95.8)
CRRT (n, %)	8 (33.3)
ECMO (n, %)	7 (29.1)
Length of ICU stay (days) (mean, SD)	47.3 (39.1)
ICU mortality (n, %)	14 (58.3)
Biological parameters	
Serum creatinine (median, IQR)	0.61 (0.47–0.79)
Albumin (g/L) (mean, SD)	2.4 (0.4)
AST (U/L) (median, IQR)	60 (28–70)
ALT (U/L) (median, IQR)	51.5 (22.102)
GGT (U/L) (median, IQR)	270 (211–309)
Total bilirubin (μmol/L) (mean, SD)	1.1 (1.2)
Mycological diagnosis *	
*Aspergillus* galactomannan antigen in BAL (median, IQR)	0.122 (0.038–0.169)
*Aspergillus* galactomannan antigen in serum (median, IQR)	0.045 (0.013–0.428)
1,3-beta-d-glucan (pg/mL) (median, IQR)	7.3 (4.3–37.9)

ICU: Intensive Care Unit; HIV: Human Immunodeficiency Virus; SAPS3: Simplified Acute Physiology Score III; CRRT: continuous renal replacement therapy; ECMO: extracorporeal membrane oxygenation; AST: aspartate-aminotransferase; ALT: alanine-aminotransferase; GGT: gamma-glutamyl-transferase; BAL: bronchoalveolar lavage; * cut-off *Aspergillus* galactomannan antigen in BAL: 0.5; cut-off *Aspergillus* galactomannan antigen in serum: 0.2; cut-off 1,3-beta-d-glucan: 7 pg/mL.

**Table 2:** Pharmacokinetic parameters estimated from the isavuconazole model.

Parameter (Units)	Mean (SE)	%RSE	CI95%	Shrinkage (%)	%CV
Volume of distribution (L)	122 (20.7)	17	81.43–162.57	16.6	67
Clearance (L/h)	5.36 (0.576)	35.7	4.23–6.49	9.6	45.3
AUC (mg·h/L)	37.31 (48.5)	-	-	-	-

AUC: area under curve; SE: standard error; RSE: relative standard error; %CV: percent covariance.

**Table 3:** Isavuconazole Cmin comparison in clinical and microbiological response and in ECMO patients.

	Clinical Response (n = 6)	Non-Clinical Response (n = 5)	*p*	Microbiological Response (n = 8)	Non-Microbiological Response (n = 3)	*p*	ECMO (n = 7)	noECMO noCRRT (n = 11)	*p*	CRRT (n = 8)	noECMO noCRRT (n = 11)	*p*
Cmin (mean (SD))	1.85 (1.22)	1.45 (0.52)	0.558	1.66 (1.09)	1.8 (0.57)	0.871	1.57 (0.63)	1.92 (0.21)	0.524	1.70 (0.40)	1.92 (0.21)	0.609
<1 mg/L (n, %)	1 (16.7)	-	-	1 (12.5)	0 (0.0)	-	2 (28.6)	1 (9.0)	-	1 (12.5)	1 (9.0)	-
>5 mg/L (n, %)	-	-	-	-	-	-	-	-	-	-	-	-

CRRT: continuous renal replacement therapy; ECMO: extracorporeal membrane oxygenation; therapeutic range 1–5 mg/L [3,20,23].

## Data Availability

Data are contained within the article.
